# Cyanides in the environment—analysis—problems and challenges

**DOI:** 10.1007/s11356-017-9081-7

**Published:** 2017-05-16

**Authors:** Ewa Jaszczak, Żaneta Polkowska, Sylwia Narkowicz, Jacek Namieśnik

**Affiliations:** Department of Analytical Chemistry, Faculty of Chemistry, Gdansk University of Technology, Narutowicza Str 11/12, Wrzeszcz, 80-952 Gdansk, Poland

**Keywords:** Cyanides in the environment, Cyanide toxicity, Cyanide determination, Cyanide in food, Tobacco smoke, Cyanide ion

## Abstract

Cyanide toxicity and their environmental impact are well known. Nevertheless, they are still used in the mining, galvanic and chemical industries. As a result of industrial activities, cyanides are released in various forms to all elements of the environment. In a natural environment, cyanide exists as cyanogenic glycosides in plants seeds. Too much consumption can cause unpleasant side effects. However, environmental tobacco smoke (ETS) is the most common source of cyanide. Live organisms have the ability to convert cyanide into less toxic compounds excreted with physiological fluids. The aim of this paper is to review the current state of knowledge on the behaviour of cyanide in the environment and its impact on the health and human life.

## Introduction

**Fig. 1:**
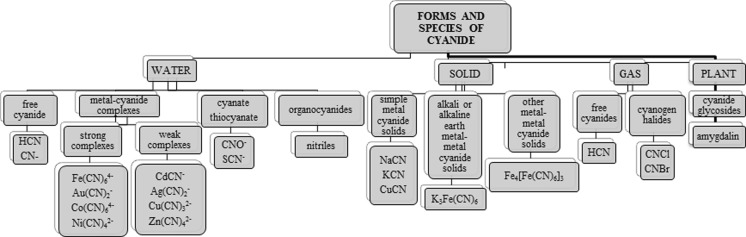
Sources of cyanide in the environment

The term “cyanides” is used to describe compounds which contain in their structure the –C≡N group. In the environment, cyanides can be found in many different forms (Kuyucak and Akcil 2013). They occur naturally in plants and processed foods. Natural sources of cyanide ions are cyanogenic glycosides which can be found in, among others, apricot kernels, cassava roots and bamboo shoots (Jones 1998). Hydrogen cyanide and cyanides are used in various industries including the mining of silver and gold. Furthermore, they are used in plastic production of all kinds of dyes as well as in chemical laboratories (Dzombak et al. 2016). The sources of environmental pollution are, among other mines, metallurgical plants and exhaust gas from vehicles. Cyanide ions get into the environment mainly from wastewater. These compounds can also enter the environment as a result of fires at industrial workshops and houses as well as from tobacco smoke (Fig. 1) (Kuyucak and Akcil 2013; Karlsson and Botz 2004; Mudder and Botz 2000; Scheneider et al. 1997).

**Fig. 2:**
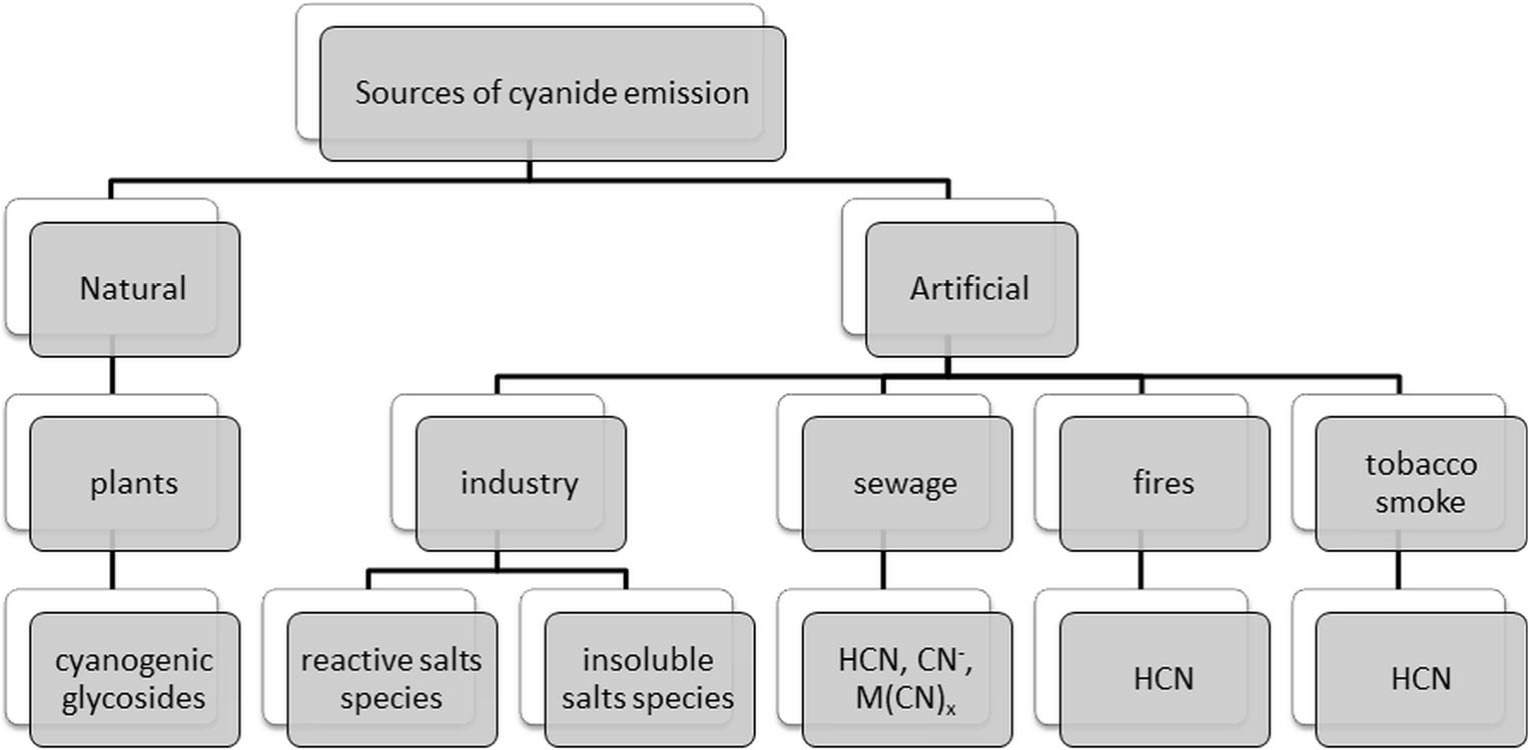
Cyanide forms and species

Their form determines their destiny within the environmental means of their transport toxicity and ecotoxicity (Fig. 2). Cyanides are present in various environmental elements such as water, soil, air exhaled, air food and biological materials like blood urine and saliva at the levels of micrograms per litre to milligrams per litre (Dzombak et al. 2016; Donald 2009). Considering the presence of cyanide in various parts of the inanimate environment and biota as well as their toxicity, there is no doubt on increasing demand for information on their prevalence in the elements of the environment or the type of material object (Dzombak et al. 2016). Based on literature data, it can distinguish a number of analytical techniques for the determination of cyanide. The most commonly used methods of cyanide ion determination are spectrophotometric techniques as well as gas and liquid chromatography (Bolstad-Johnson et al. 2000). This review examines the current state of knowledge on the behaviour of cyanide ion in the environment.

## Cyanide occurrence in the environment

### Atmosphere

In air, cyanide ions are present mainly as hydrogen cyanide (HCN). Miners, firefighters and workers of metallurgical chemical and galvanic industries are exposed largely to cyanide poisoning (Bolstad-Johnson et al. 2000). Cyanides enter into the atmosphere as a consequence of industrial processes and fires at houses and industrial halls. Hydrogen cyanide is a product of combustion of synthetic polymers, wool and silk; additionally, it is produced during the combustion of fuels in automobile engines as a result of catalytic reduction of nitrogen oxides. However, the concentration of HCN in the exhaust gas is higher only in the absence of catalyst (Karlsson and Botz 2004). Cyanide ions are generated naturally during biogenic processes of higher plant bacteria and fungi (Mudder and Botz 2000).

**Fig. 3:**
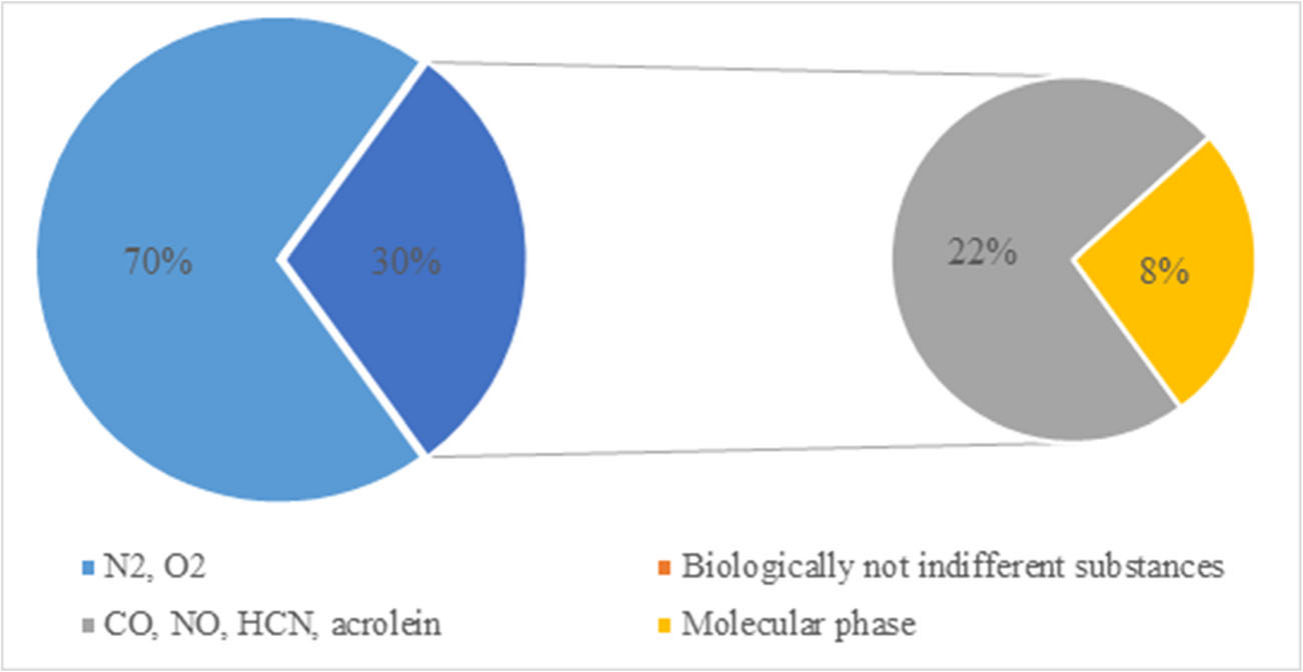
Tobacco smoke components

Analysis of data presented in the literature leads to the conclusion that smoking and, as a result of it, tobacco smoke are the most significant source of cyanide emissions to the air (Table 1). In tobacco smoke, which is formed during smoking, two types of stream can be distinguished: the main and the side ones. Tobacco smoke has 400–500 chemical components of the gas phase and 3500 components of condensed phase. Hydrogen cyanide is a part of the biologically not indifferent substances, which account for about 22% of 500 mg of smoke inhaled from a single cigarette by the smoker (Fig. 3). Hydrogen cyanide is formed in the burning area, mainly during the pyrolysis of various nitrogen compounds, such as proteins and nitrates, at a temperature higher than 700 °C and with oxygen deficit (Borgerdinga and Klusb 2005). In the air, cyanides occur mostly in gaseous form and can be transported over long distances from the emission source (Petrova Simenova and Fishbein 2004). The duration of hydrogen cyanide in the atmosphere is estimated to be approximately 5 months (Karlsson and Botz 2004; Scheneider et al. 1997).

**Table 1:** Literature information on cyanide concentrations in different environmental samples

Type of sample	Source of sample	Concentration	References
Air			
Outdoor air	Lower atmosphere	0.36 ± 0.16 ppbv	Ambose et al. (2012)
	Atmosphere	333 ± 44 pptv (summer)	Zhao et al. (2000)
		195 ± 16 pptv (winter)	
	Lower stratosphere	233.5 ± 160.6 ppt	Singh et al. (2003)
		280 ± 4 pptv	Viggiano et al. (2003)
	Stratosphere	164 pptv	Scheneider et al. (1997)
	Gold field	0.76 ppb	Orloff et al. (2006)
	Vehicular emissions	654 t/year	Moussa et al. (2016)
	Vehicular emission	0.45 mg/km	Karlsson and Botz (2004)
Indoor air	Vehicular exposure in garage	0.32 μg/m^3^	Karlsson and Botz (2004)
	Air in car	14–20 ppm	Mangnusson et al. (2012)
	Fire	1.8 ± 3 mg/kg	Paton-Walsh et al. (2010)
Tobacco smoke			
Cigarette	China	125.2 μg/cig.	Zhang et al. (2011)
	Spain	6.6 μg/ cig.	Marcilla et al. (2012)
	Russia	27 μg/cig.	Ashley et al. (2014)
	CAMEL Lights	184.825 μg/cig.	Mahernia et al. (2015)
	Marlboro Gold (Germany)	165.871 μg/cig.	
	Marlboro Extra (USA)	164.309 μg/cig.	
	Marlboro Lights (Switzerland)	69.344 μg/cig.	
	Winston Blue (Europe)	99.244 μg/cig.	
	Switzerland	4.1 ng/cig.	Mottier et al. (2010)
	China	98.38 μg/cig.	Xu et al. (2006)
Water			
Surface water	Korea (Gum River)	1.01 ± 0.03 μg/L	Kang and Shin (2014)
	–	0.77 mg/L	Dadfarnia et al. (2007)
	Brazil	25–50 μg/L	Frizzarin and Rocha (2013)
	China	–	Wan et al. (2015)
	Italy	5.11 μg/L	Giuriati et al. (2004)
Drinking water	USA (Sunnyvale)	<LOD	Christinson and Rohrer (2007)
	USA (San Jose)	<LOD	
	Sweden	–	Themelis et al. (2009)
	Iran	<LOD	Absalan et al. (2010)
Tap water	Iran	0.6 μg/L	Abbasi et al. (2010)
Wastewater			
	Petrochemical sludge	6.1–63.5 μg/L	Dadfarnia et al. (2007)
	Electroplating waste	0.04–1.2 μg/mL	Hassan et al. (2007)
	Petrochemical sludge	4600.2 μg/L	Abbasi et al. (2010)
	Gold cyanidation solution	540 mg/L	Breuer et al. (2011)
	Industrial wastewater	–	Noroozifar et al. (2011)
Soil			
	Japan	0.060 mg/L	Matsumura and Kojima (2003)
	Coking plant sites (Germany)	32.8 ± 1.44 mg/kg	Mansfeldt and Biernath (2000)
	Coking plant sites (France)	46.5 ± 14.5 mg/L	Manar et al. (2011)
	Goldmine (Tawurbiek, China)	70.55 μg/g	Shehong et al. (2005)
	Coking plant sites (Germany)	0.14 mg/L	Rennert and Mansfeldt (2006)
	Gold mine (Brazil)	0.83–1.44 mg/kg	Prereira and Sousa Neto (2007)
	Techatticup Mine site (USA)	<0.01 mg/kg	Sims and Francis (2008)
Fresh food			
Kernel/seed	Apple	2.80 ± 0.02 mg/kg	Ma et al. (2010)
		690 ppm	Haque and Bradbury (2002)
		1–3.9 mg/g	Bolarinwa et al. (2015)
	Apricot	1.88 ± 0.07 mg/kg	Ma et al. (2010)
		785 ppm	Haque and Bradbury (2002)
		14.37 ± 0.28 mg/g	Bolarinwa et al. (2014)
	Peach	710 ppm	Haque and Bradbury (2002)
	Nectarine	196 ppm	
	Plum	696 ppm	
	Bean	1.76–1.77 mg/kg	Chove and Mamiro (2010)
	Millet	2.11–2.14 mg/kg	
	Lensed	390 ppm	Haque and Bradbury (2002)
	Rubber tree	–	Abdullah et al. (2013)
	Nuts	–	Chove and Mamiro (2010)
	Plum	247 mg/100 g	Surleva and Drochioiu (2013)
	Almond	7.4 μg/100 g	
	Apple	108 mg/100 g	
	Flax	7.3 mg/100 g	
Leaf	Sorghum	750 ppm	Haque and Bradbury (2002)
	*Alocasia macrorrhizos*	29 ppm	
	Spinach	2.51 ± 0.6 μg/g	Kuti and Konoru (2006)
		1.28 ± μg/g	
	Chokecherry	4.7–15 mg/kg	Pentore et al. (1996)
	Bamboo	1010 ppm	Haque and Bradbury (2002)
	Grapevine	123–329 mg/kg	Franks et al. (2005)
Root	Manioc	27 ppm	Haque and Bradbury (2002)
Processed food			
Liquor	Cherry	1 ng/mL	Wu et al. (2015)
Juice	Apple juice	0.003 mg/mL	Bolarinwa et al. (2015)
Marzipan		0.02 mg/g	Bolarinwa et al. (2014)
Flour	Manioc	43 ± 20 ppm	Haque and Bradbury (2002)
		232 ± 10 mg/kg	Tivana et al. (2014)
		2.3 mg/kg	Kalenga Saka and Nyirenda (2012)
	Garri	16.7 ppm	Bradbury (2009)

### Water

There are known many emission sources of cyanides to surface waters. Cyanides can contaminate the water through discharges of factory wastes and can be washed down from fields and urban areas. As a component of wastewater, they are present in the effluents from electroplating processes, gold and silver extraction and production of medicines and plastic (Table 1) (Barclay et al. 1998; Dursun and Aksu 2000).

Water containing cyanide ions is often treated with sulphur dioxide, chlorination process and/or aeration. The most efficient method uses Caro’s acid (hydroperoxysulphuric acid). Techniques based on chlorination are effective only for free cyanides and weak metal complexes. Other methods, such as ozonation or reverse osmosis, are very expensive or inefficient. Biological treatment is possible thanks to microorganisms, such as fungi (e.g. *Fusarium solani*) and bacteria (e.g. *Pseudomonas fluorescens*). In aerobic conditions and with the presence of glucose, microorganisms use ferrocyanide as a source of nitrogen and carbon. As a result of both aerobic and anaerobic biodegradability, ammonia, carbon dioxide and formates are formed. The best conditions for maximum biodegradability of cyanide ions were observed with a glucose concentration of 0.0465 g/L and pH = 5 (Barclay et al. 1998; Dursun and Aksu 2000).

### Soil

The presence of cyanide ions in the soil is primarily caused by such anthropogenic manifestation as galvanic and metallurgical industry (Table 1). The waste containing high concentrations of cyanide is produced also during the underground coal gasification. The degree of contamination of soil with cyanides depends on their amount and activity. Most of cyanides are deposited in the environment as complexes of Fe(CN)_6_^3−^ and Fe(CN)_6_^4−^. Their toxicity is low, but due to the light, they convert into highly toxic and volatile free cyanides. In soil, without the light, this process is very slow (Meeussen et al. 1995). This can be described by the following reaction:

1. Decomposition of ferrocyanide to less toxic ferricyanide

$$ \mathrm{Fe}{\left(\mathrm{CN}\right)}_6^{4-}+ hv\to \mathrm{Fe}\ {\left(\mathrm{CN}\right)}_6^{3-}+{\mathrm{CN}}^{-} $$

2. However, due to the light, they decompose into volatile and highly toxic hydrogen cyanide

$$ {\mathrm{Fe}\left(\mathrm{CN}\right)}_6^{3-}+{6\mathrm{H}}_2\mathrm{O}+{3\mathrm{H}}^{+}\leftrightharpoons {\mathrm{Fe}\left(\mathrm{OH}\right)}_3\left(\mathrm{s}\right)+6\mathrm{H}\mathrm{CN}\left(\mathrm{aq}\right) $$

**Fig. 4:**
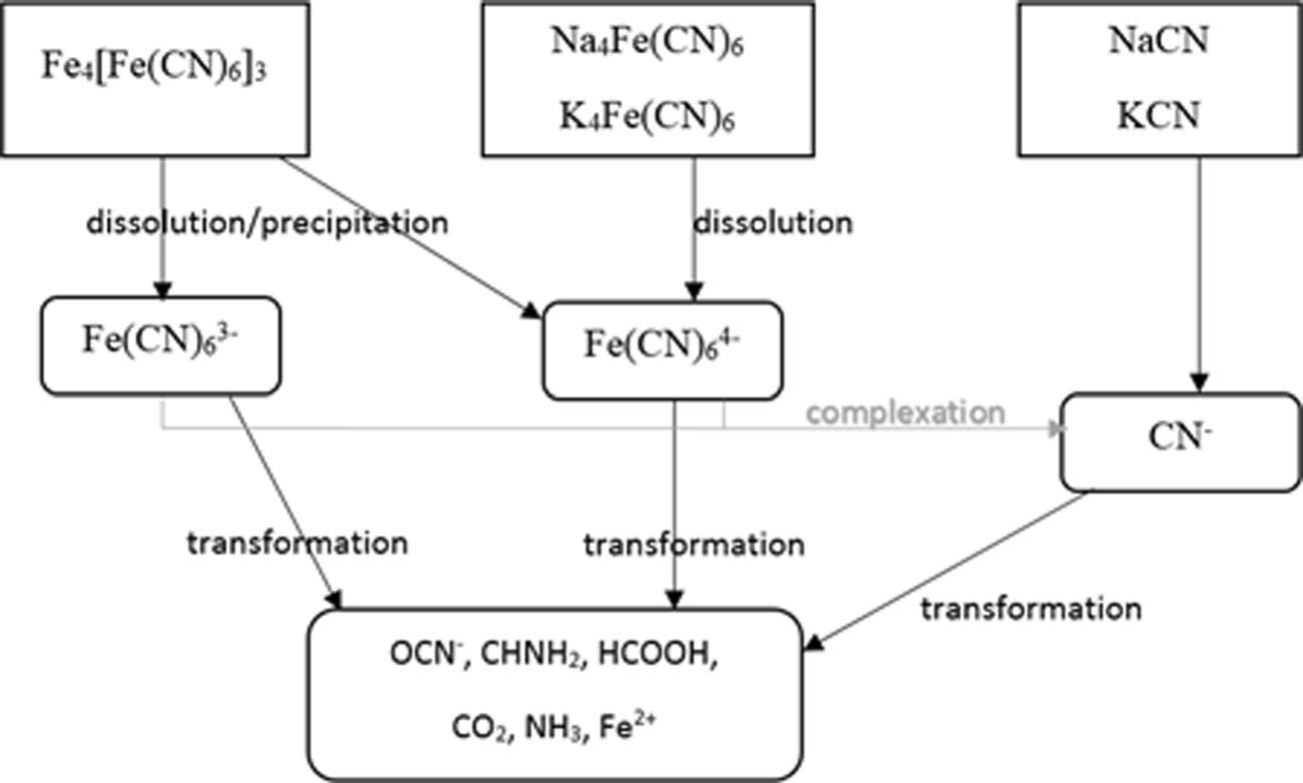
Processes of cyanide transformations occurring in water and soil

3. Cyanide ions in the soil undergo many transformations (Fig. 4), and the result of soil contamination with cyanides is its blue coloration, derived from Fe_4_[Fe(CN)_6_]_3_, i.e. iron ferrocyanide, known also as Prussian blue when its concentration is 100–500 mg CN/kg (Shifrin et al. 1996).

### Food

The reason for cyanide poisonings, as a consequence of food consumption, is cyanogenic glycosides in plants (Table 1). The most common cyanogenic glycoside is amygdalin that can be found in seeds, pips and kernel of fruit such as apples, peaches, almonds, cherries, plums and apricots (Table 2). The amount of amygdalin in processed products is lower than that in the seeds (Donald 2009). The level of toxins depends on growing conditions, such as climate, and consumed parts of the plant (Kuti and Konoru 2006; Haque and Bradbury 2002).

**Table 2:** Plants containing cyanogenic glycosides

Plant	Genera and species	Main cyanogenic glycosides	Literature
Grain crops	Wheat (np. *Triticum monococcum*) Hordeum *(Hordeum vulgare*) Avena (*Avena sativa*) Secale (*Secale cereale*) Sorghum (*Sorghum bicolor*) Millet (*Eleusine coracana*)	Cycasin Vicianin Sambunigrin Dhurrin	Vetter (2000)
Vegetables	Bean (*Phaseolus lunatus*) Manioc (*Manihot esculenta*) Taro (*Colocasia esculenta*) Spinach (*Cnidoscolus aconitifolius*)	Linamarin Linustatin Lotaustralin	Ballhorn (2011)
Fruit	Apple (*Malus pumila*) Peach (*Prunus persica*) Nectarine (*Prunus persica* var. *nucipersica*) Apricot (*Prunus armeniaca*) Bamboo Shoots (*Bambusa arundinacea*) Plum (*Prunus* sp.) Almond	Prunasin Amygdalin	Senica et al. (2016)

A common cause of cyanide poisoning is unconscious consumption of large quantities of poorly processed foods such as cassava. In manioc, one of the main crops in tropical regions, linamarin is present only in bitter variety. At the same time, a variety of sweet manioc is safe for direct consumption, and it is obtained after rinsing several times the bitter one. The result was the loss of water-soluble glycosides (Bradbury et al. 2011; Cumbana et al. 2007). In Italy, cherries with pits are used for home-made tinctures (Pentore et al. 1996). In the Southeast Asia, sodium cyanide (NaCN) is still used as a method for fishing (Mak et al. 2005).

**Fig. 5:**
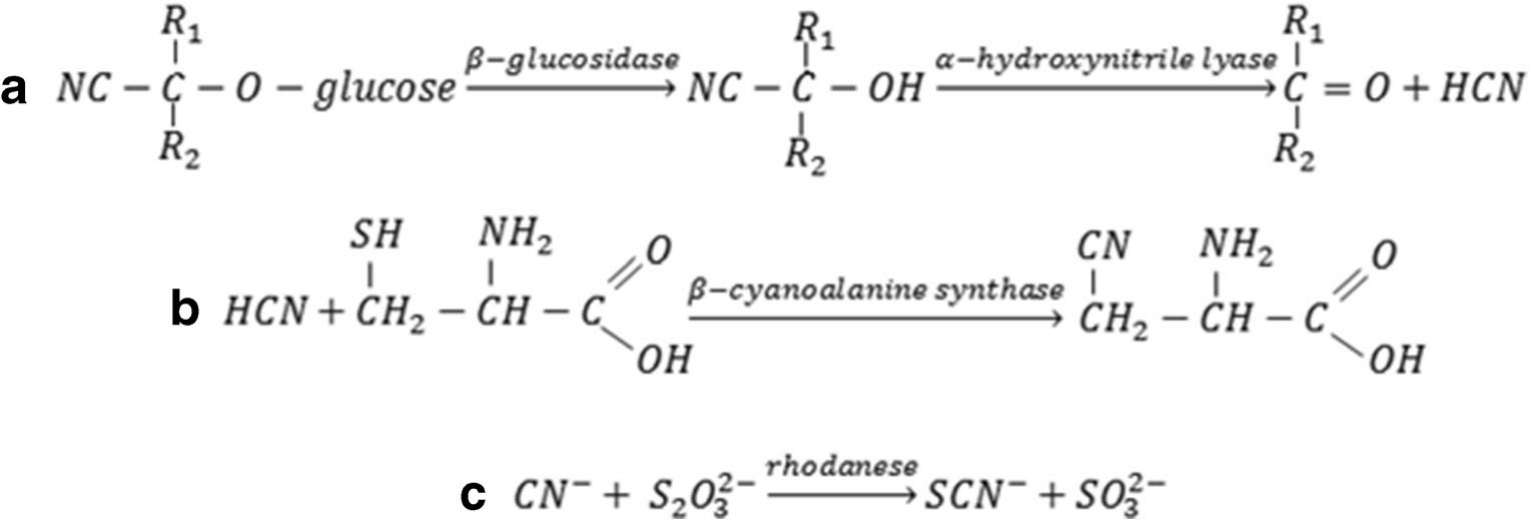
Catabolism and detoxification of cyanogenic glycosides

In plants, metabolism of cyanides involves β-cyanoalanine formation due to reaction of hydrogen cyanide with cysteine. Then, β-cyanoalanine is transformed into asparagine (Fig. 5) (ATSDR 1997; Zagrobelny et al. 2004). An example is amygdalin—its decomposition inside the body due to enzymatic hydrolysis is initiated by the enzyme β-glucosidase, and it results in the suitable α-hydroxynitrile, which, at pH values above 6, dissociates into sugar, ketone and hydrogen cyanide (Fig. 5). At lower pH values, the reaction is catalysed by α-hydroxynitrile lyase.

### Cyanides in biological materials

As a result of various industrial activities and lifestyle, cyanide ions are introduced into the human body. Biological materials are an excellent source of information on environmental pollution and its impact on human health (Ballantyne 1983).

Urine and saliva are frequently selected as biological materials for research (Table 3) due to the fact that both can be sampled in non-invasive way, and also, the size of the sample fluid is relatively large (Sano et al. 1989a, b). Another commonly used material is blood, where cyanide determinations can be performed, as well as adducts with proteins and their metabolites. The half-life of cyanide ions in the body is about 2 h; so, to often assess the exposure on the tobacco smoke components, thiocyanate ions are used as their half-life in the body is approx. 6 days (Narkowicz et al. 2013a, 2015). Elevated concentrations of cyanides in the blood can be fatal. In case of death in fire, the results of toxicological studies of the victims, such as the level of carboxyhaemoglobin and cyanide concentration level in the blood, can be used to determine the origins and type of fire (McAllister et al. 2008).

**Table 3:** Literature information on cyanide concentrations in biological samples

Type of sample	Source of sample	Concentration	References
Liquid			
Blood	Poisoning	2.77 mg/L	Sanchez-Verlaan et al. (2011)
	Fire victims	1.06 mg/L	McAllister et al. (2011)
	Fire victims	2.0–7.2 mg/L	Ferrari et al. (2001)
	Fire victims	1.06 mg/L	Yeoh and Braitberg (2004)
	Health volunteers	0.08 μM/mL	Kage et al. (1996)
	Fire victims	5.32 mg/L	Moriya and Hashimoto (2003)
	Post-mortem blood sample	0.03 mg/L	Felby (2009)
	Living organism	0–0.04 mg/L	
Urine	Health volunteers	0.1 mg/L	Cruz-Landeira et al. (2000)
	Death poisoning	0.15 g/mL	Liu et al. (2009a)
	Smoker	518 ± 123 nM	Zhang et al. (2015)
	Smoker	0.42 μM/L	Jermak et al. (2006)
	Non-smoker	n/a	
	Non-smoker volunteers	0.15 μg/mL	Liu et al. (2009b)
Nasal discharge	Health volunteers	0.121 mg/L	Narkowicz et al. (2013b)
Saliva	Health volunteers	0.66 ± 0.52 μM	Tsunge et al. (2000)
	Smoker	0.76 μM/L	Jermak et al. (2006)
	Non-smoker	0.38 μM/L	
Plasma	Non-smoker volunteers	11.4 μg/mL	Liu et al. (2009b)
Gastric content	Suicide victim	135 μg/mL	Minakata et al. (2009)
Gas			
Breath	Health volunteers	14 ppb	Španěl et al. (2007a)
	School students	7 ppb	Španěl et al. (2007b)
	Three volunteers	0–62 ppbv	Ma et al. (2010)
	Patients with lung disease	25.1 ppb	Dummer et al. (2013)

## Metabolism of cyanide

**Fig. 6:**
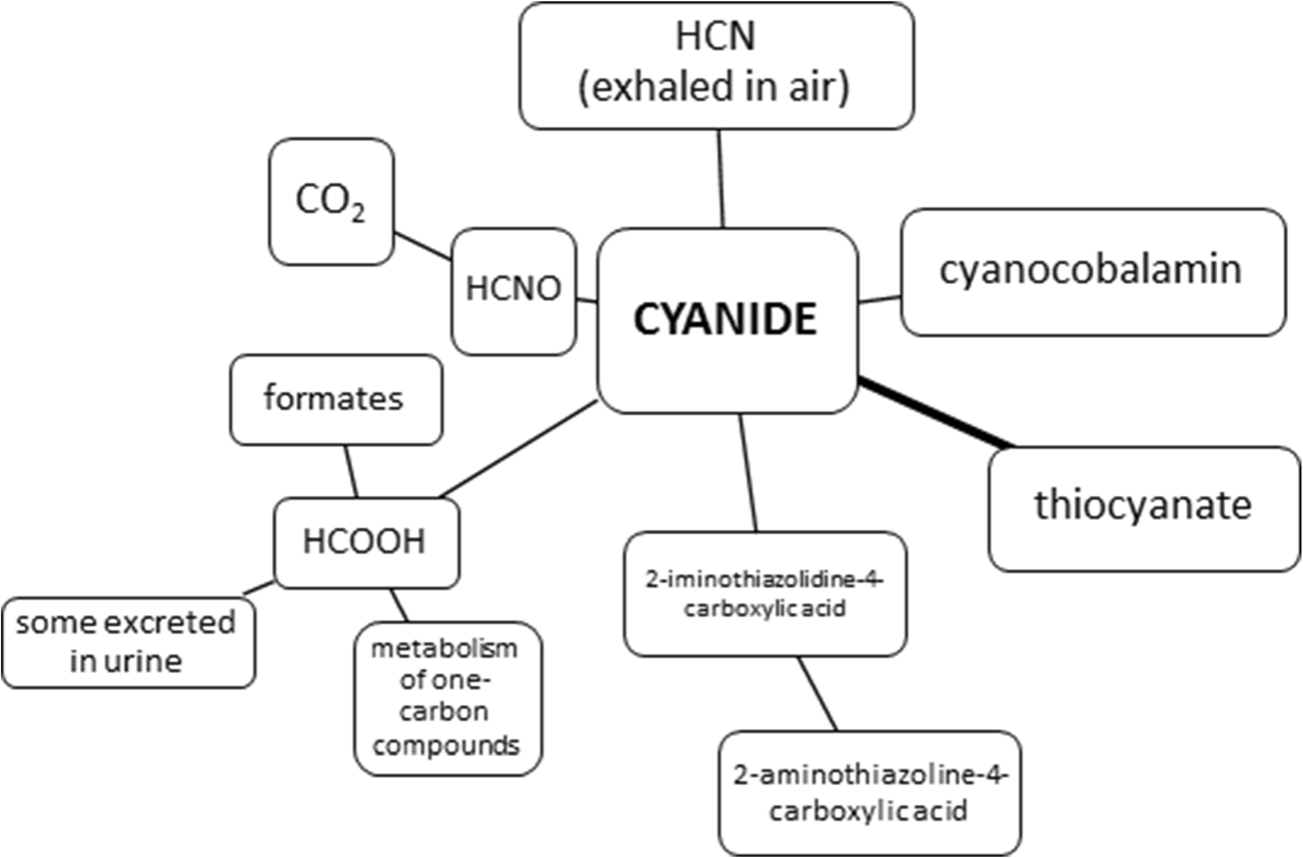
Basic processes involved in the metabolism of cyanide

As results of pollutions, cyanides get into the environment and they can negatively affect living organisms in many ways (Abraham et al. 2016). The cyanide anion is absorbed easily, by the mucous membrane of the respiratory tract, through the skin especially the wet one and gastrointestinal tract. In case of animals, hydrogen cyanide reacts with methaemoglobin in the bloodstream; however, most of cyanide metabolism occurs in tissues. A substantial part (80%) of cyanides is a subject to detoxification in the liver. Responsible for it is thiosulphate sulphutransferase enzyme present in the mitochondria of the liver. Sulphur which is required for this reaction is collected from biological compounds such as, for example, thiosulphates (Fig. 5). As a consequence of this reaction, thiocyanate ions are formed and they are approximately 200 times less toxic than cyanide excreted with body fluids. The process of cyanide metabolism in a living organism can occur in various ways (Fig. 6), among others, as a combination of cyanide with vitamin B_12a_ resulting in cyanocobalamin, i.e. vitamin B_12_ (Petrova Simenova and Fishbein 2004). The rest of cyanides are oxidized to formate and carbon dioxide. Formates are excreted into urine while carbon dioxide, along with hydrocyanic acid, by the lungs. In the small amount, cyanides react with cysteine to form 2-iminothiazolidine-4-carboxylic acid (Petrova Simenova and Fishbein 2004).

## Cyanide toxicity for a living environment

**Fig. 7:**
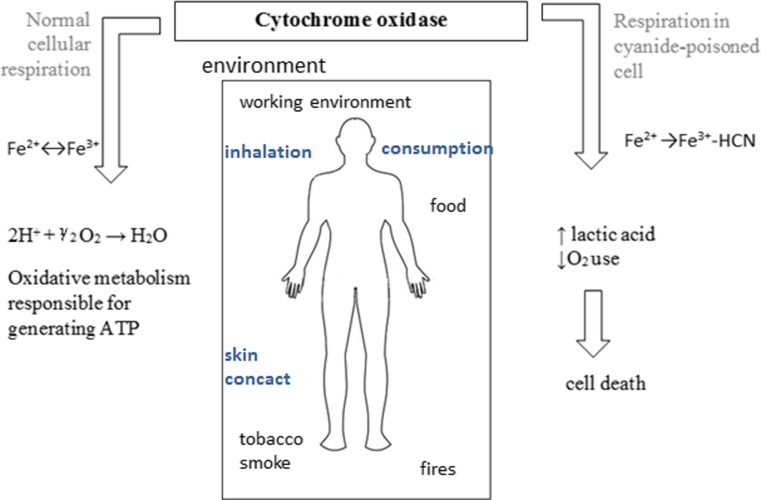
Schematic representation of the impact of cyanides on the human body

Compounds containing cyanide ions are rapidly acting poison, as they disrupt the process of cellular respiration. The basic effect of cyanide activity involves combining with trivalent iron of cytochrome oxidase, which is a key enzyme of the respiratory chain (Fig. 7). This combination results in blocking of the intracellular respiratory and increasing synthesis of lactic acid. Although the blocking of cytochrome oxidase has the most significant impact, it ought to remember that the CN^−^ ions also inhibit other enzymes: glutamate decarboxylase, xanthine oxidase, superoxide dismutase, NO synthase and nitrite reductase. Cyanide ion can cause direct damage to the nervous system by lipid peroxidation (Sun et al. 1995). Most sensitive to toxic effects of cyanides are tissues with the fastest metabolism of oxygen, so the brain and the heart muscle, but hypoxia causes the disorder of all body cells’ functioning.

**Fig. 8:**
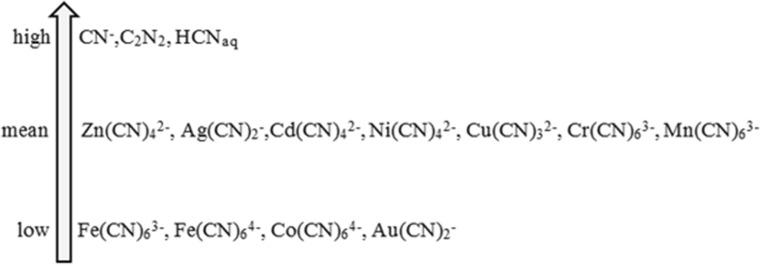
Toxicity of cyanide forms

A toxic dose depends largely on the type of compound containing a cyanide ion. Based on the data presented in the literature, it can be concluded that the toxicity of cyanides largely depends on the form of their occurrence (Fig. 8). The least toxic are complex cyanide compounds in contrast to free ions, which are the most toxic ones (Johnson 2015; Donato et al. 2007).

Cyanide toxicity (Table 4) is a parameter which defines the scope of their application. Numerical values for LC_50_ and LD_50_ are generally determined after 24-h exposure of the body to a predetermined dosage or concentration of the compound containing a cyano group. The most commonly used indicator organisms are daphnia (*Daphnia magna*) as well as fishes, mice and rats.

**Table 4:** Cyanide toxicity

Organism	Cyanide form	Parameter/exposure time	Concentration range	References
Fish				
*Oncorhynchus mykiss*	K_3_Co(CN)_6_	LC_50_/96 h	112.9 mg/L	Little et al. (2007)
*Cyprinus carpio*	NaCN	LC_50_/96 h	1.0 mg/L	David and Kartheek (2016)
*Penaeus monodon*	NaCN	LC_50_/96 h	0.110 mg/L	Pablo et al. (1997a, b)
	K_3_Fe(CN)_6_		9.1 mg/L	
	K_4_Fe(CN)_6_		60.8 mg/L	
*Salmo gairdneri*	HCN	LC_50_/96 h	0.057 mg/L	McGeachy and Leduc (1988)
*Cyprinus carpio*	NaCN	LC_50_/96 h	1.0 mg/L	David et al. (2008)
*Acanthopagrus butcher*	NaCN	LC_50_/96 h	70 μg/L	Pablo et al. (1996)
	K_3_Fe(CN)_6_	LC_50_/96 h	1730 μg/L	
	K_4_Fe(CN)_6_	LC_50_/96 h	20.5 μg/L	
*Carassius auratus*	NaCN	LC_50_/96 h	318 μg/L	Cardwell et al. (2006)
*Lepomis macrochirus*	NaCN	LC_50_/96 h	134 μg/L	Kimball et al. (1978)
	HCN	NOEC	<5 μg/L	
*Macquaria novemaculeata*	NaCN	LC_50_/96 h	109 μg/L	Pablo et al. (1996)
	K_3_Fe(CN)_6_	LC_50_/96 h	2830 μg/L	
	K_4_Fe(CN)_6_	LC_50_/96 h	285,000 μg/L	
*Pimephales promelas*	NaCN	LC_50_/8 days	114 μg/L	Cardwell et al. (2006)
*Pimephales promelas*	HCN	NOEC-LOEC/256 days	12.9–19.6 μg/L	Lind et al. (1977)
Invertebrates				
*Daphnia magna*	NaCN	LC_50_/24 h	0.171 mg/L	Jaafarzadeh et al. (2013)
		LC_50_/48 h	0.12 mg/L	
		LC_50_/72 h	0.07 mg/L	
		LC_50_/96 h	0.019 mg/L	
*Daphnia magna*	K_3_Co(CN)_6_	LC_50_/96 h	0.502 mg/L	Little et al. (2007)
*Ceriodaphnia dubia*		LC_50_/96 h	2.289 mg/L	
*Chlamys asperrimus*	NaCN	EC_50_/48 h	0.0286 mg/L	Pablo et al. (1997a, b)
	K_3_Fe(CN)_6_		0.128 mg/L	
	K_4_Fe(CN)_6_		0.686 mg/L	
*Asellus communis*	HCN	NOEC-LOEC/112 days	29–40 μg/L	Oseid and Smith (1979)
*Gammarus fasciatus*	NaCN	LC_50_/96 h	900 μg/L	Ewell et al. (1986)
*Cyclops viridis*	NaCN	LC_50_/96 h	158 μg/L	Sarkar (1990)
Algae				
*Nitzschia closterium*	NaCN	EC_50_/72 h	57 μg/L	Pablo et al. (1997a, b)
	K_3_Fe(CN)_6_	EC_50_/72 h	127 μg/L	
	K_4_Fe(CN)_6_	EC50/72 h	267 μg/L	
*Scenedesmus quadricauda*	KCN	LOEC/8 days	30 μg/L	Bringmann and Kühn (1980)
*Pseudokirchneriella subcapitata*	NaCN	EC_50_/72 h	116 μg/L	Manar et al. (2011)
	K_3_Fe(CN)_6_	EC_50_/72 h	158 μg/L	
	K_4_Fe(CN)_6_	EC_50_/72 h	283 μg/L	
Upper organism				
Mice	KCN	LD_50_/24 h	8.4 mg/kg	Yamamoto (1995)
Mice	KCN	LD_50_/24 h	8.87 mg/kg	Jiang et al. (1998)
Rat	CH_3_CN	LD_50_/24 h	>5000	Rao et al. (2013)
	CH_2_CHCN		95.1 mg/kg	
	CH_2_(CN)_2_		66.4 mg/kg	
	CH_3_CH_2_CN		83.6 mg/kg	
	Na_2_[Fe(CN)_5_NO]·2H_2_O		83.6 mg/kg	
	C_2_H_4_(CN)_2_		378.5 mg/kg	

The estimated lethal dose for an adult human is 1.5 mg CN^−^kg of body weight. Symptoms of severe poisoning by inhalation are observed from 53 mg HCN/m^3^, while the lethal dose ingested with food is approx. 200–300 mg (Oluwole et al. 2003). Prolonged exposure to cyanide can lead to body weakness and various diseases such as hypothyroidism, kidney damage and miscarriages (Table 5).

**Table 5:** Cyanide poisoning symptoms; Akintonwa et al. 1994)

	Symptoms
Nervous system	Headache, agitation, seizures, coma, mydriasis
Respiratory system	Shortness of breath, cough
Cardiovascular system	Sudden cardiac arrest, acute coronary syndrome, pulmonary oedema, supraventricular and ventricular arrhythmias
Digestive system	Abdominal pain, nausea, vomiting
Skin	Cherry-red colour of the skin, excessive sweating

## Determination of cyanide in different types of samples

Cyanide ions have a toxic effect on the health and safety of people. Biological materials collected from humans provide researchers with information regarding the health and may also be used to define the environmental pollution. Therefore, it is necessary to determine their content in representative samples taken also from inanimate objects of the environment.

### Problems and challenges posed by the analysis of cyanide in environmental and biological samples

Stages of sampling, preservation and storage are crucial for the analysis of the presence of cyanide. In case of biological samples, storage temperature of samples is very important as it may change the cyanide ion concentration up to 66% (Lindsay et al. 2004).

A number of analytical challenges can occur while examining environmental samples and biological materials on the amount of cyanides, and they ought to be taken into account at the stage of developing and implementing new analytical procedures to the current ones (Narkowicz et al. 2012) (Table 6).

**Table 6:** Analytical challenges in the development of new analytical procedures

Regardless of used analytical procedures
- Heterogeneity of environmental samples and biological materials - Metabolism of cyanide depends on the age and sex of the donor - Small volume of samples - Losses of cyanide during sampling and sample preparation - The use of reducing agents - The presence of interferents
Related to the analytical procedure
- Stage of sample preparation depends on the applied analytical technique - A complex composition of the matrix - The ability to change the sample components while collecting, storing and transporting samples - Low concentrations of cyanide ions - The possibility of reaction between the compounds present in samples - Oxidizers can co-exist with cyanide

**Fig. 9:**
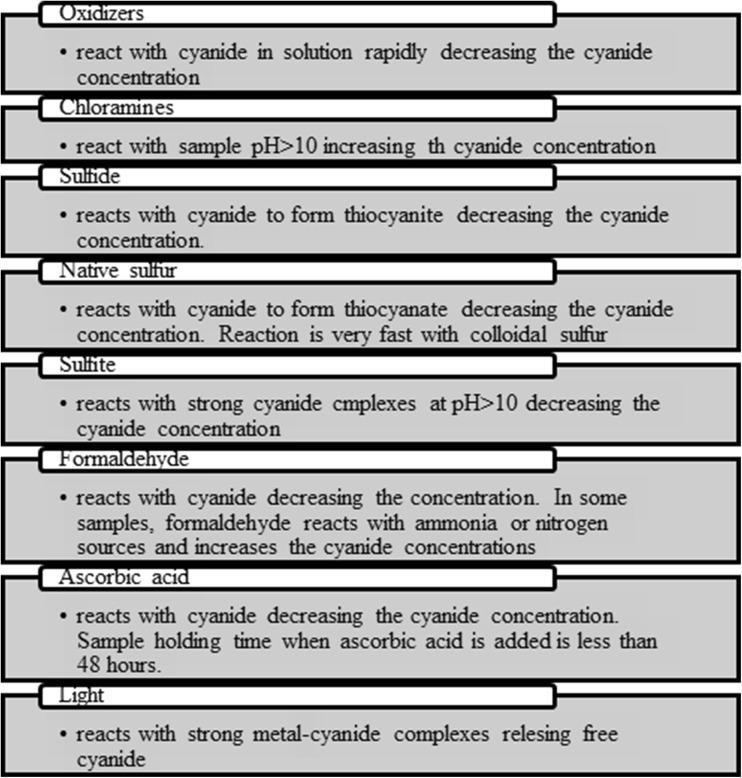
Interfering substances in the determination of cyanide ions

Samples of biological material, wastewater and food are complex matrix ones, as they require adequate preparation for analysis (Table 7). Interferents present in the sample can react with cyanide; thus, they contribute to the errors in the results of analysis (Fig. 9). During preparation of the sample for analysis, in the extraction step beside decomposition of stable metal cyanide complexes, elimination of interfering substances occurs (Christinson and Rohrer 2007; ASTM D 7365-09a 2015).

**Table 7:** The composition of the matrix of environmental and biological samples

Sample	Example	Constituents of the matrix	References
Food	Almond	Linoleic acid, elaidic acid	Lin et al. (2016)
Water	Drinking water	CaCO_3_, Ca, Cl^−^, PO_4_^3−^, Fe^2+^, Mn^2+^	Gerke et al. (2016)
Air		Benzene, CO, Pb, NO_*x*_, PM_10_, SO_2_, VOCs	DOE (1997)
Soil	Calabria, Italy	SiO_2_, TiO_2_, Al_2_O_3_, FeO, MgO, CaO, Na_2_O, K_2_O, Cl_2_O	Pelle et al. (2013)
Tobacco smoke		Benzo[a]pyrene, HCN, formaldehyde acetaldehyde, acrolein, benzene, toluene	Torikaiu et al. (2005)
Biological samples	Blood	Erythrocytes, leukocytes, protein, haemoglobin (HGB), neutrophils, blood platelets, glucose	Tong et al. (2009)
	Urine	Na^+^, K^+^, NH^3+^, Ca^2+^, Mg^2+^, Cl^−^, PO_4_^3−^, SO_4_^2−^, water, urea, uric acid, creatinine	Yaroshenko et al. (2015)
	Saliva	Salivary amylase (ptyalin) and maltase Mucin—mucilaginous body Cl^−^, NO_3_^−^, SO_4_^2−^, SCN^−^, protein, water 99.5%	Chen et al. (2015)

### Sample preparation stages

**Fig. 10:**
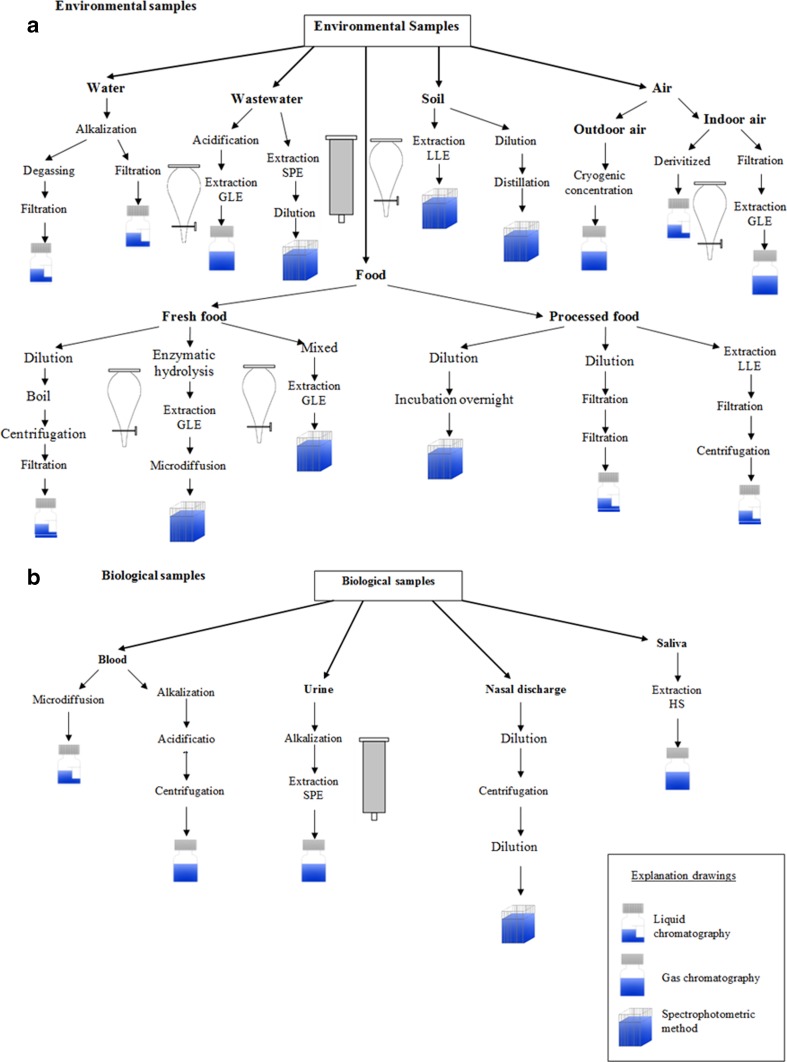
**a**, **b** Sample preparation

Preparation of the sample for analysis usually involves adding basic reagents and extracting cyanide from the sample (Fig. 10a, b). In environmental samples as well as in biological ones, it is necessary to add sodium hydroxide to stabilize the form that cyanide occurred in. The addition of NaOH results in a sample with pH above 11, and as consequence, volatile forms of cyanide are bound. Hydrogen cyanide is formed in solutions of cyanide ion complexes with metals at pH below 4. Distillation of the sample with strong acid causes the release of hydrogen cyanide but prevents determining it as free cyanide.

In order to prepare the plant samples to be analysed for the presence of cyanide, firstly, parts of plants for examination need to be thoroughly washed in distilled water and then dried for 24 h, after grinding. Later on, the extraction is carried out with NaOH or H_3_PO_4_. While determining cyanogenic glycosides, three gradual enzymatic biodegradations are required. For example, in case of amygdalin in the first step, it is necessary to separate it from prunasin and glucose. The second step is hydrolysis of prunasin to mandelonitrile and glucose. In the final phase of hydrolysis, mandelonitrile decomposes to benzaldehyde and hydrogen cyanide. Enzymatic hydrolysis of amygdalin to mandelonitrile usually takes place under mild acid conditions at a pH of 5–5.8, whereas the hydrolysis of mandelonitrile to benzaldehyde and HCN takes place quickly under basic conditions (at pH10) (Ma et al. 2010; Bolarinwa et al. 2015).

In sample analysis, preparing samples is an extremely important stage, including the case of biological samples with important information, like during post-mortem examination. Looking at blood samples, it is necessary to separate cyanide ions from haemoglobin, and it can be achieved among others by microdiffusion in the Conway cell (Gambaro et al. 2007). In order to improve the efficiency and accuracy of the analytical techniques, researchers use fibre-protected headspace liquid-phase microextraction or solid-supported liquid-liquid extraction combined with capillary electrophoresis (Mak et al. 2005).

**Fig. 11:**
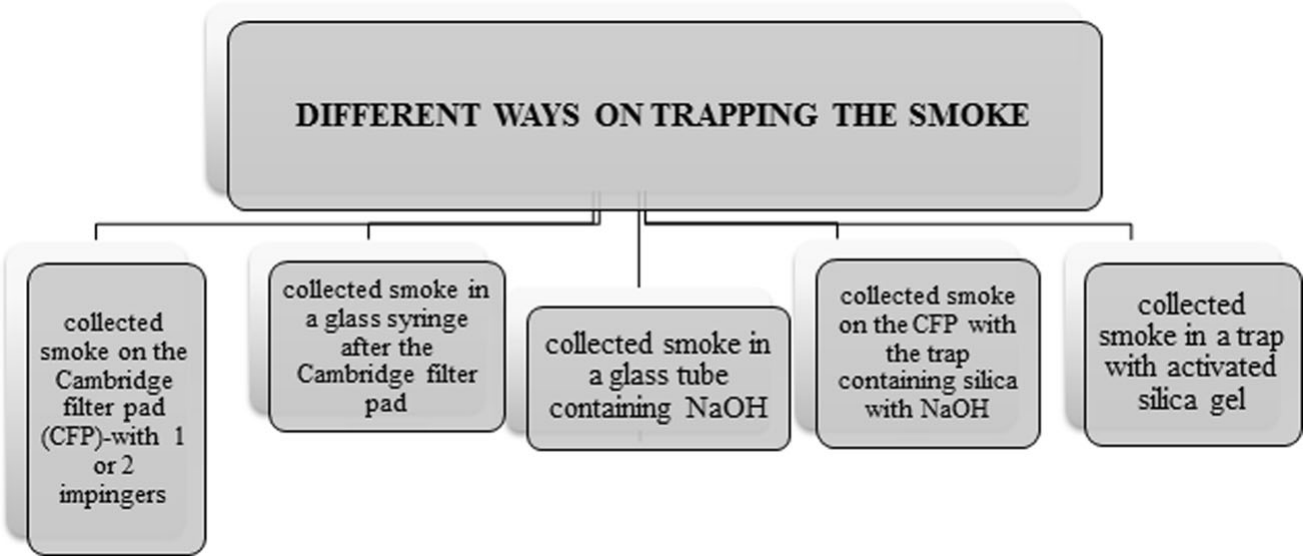
Information on the use of traps to trap tobacco smoke constituents

The tobacco smoke contains over 5600 compounds, which means that cyanide determination is a very complicated process (Thorne and Adamson 2013). For sampling smoke, special apparatus is used to simulate the process of cigarette smoking by man. They adjust the number of puffs per minute and puff volume. Moreover, such devices are equipped with pumps, flow meters and traps with capture solution to trap components of the tobacco smoke (Fig. 11) (Mahernia et al. 2015; Intorp et al. 2008).

### Analytical techniques for determining cyanide in different samples

The most common analytical techniques used for detection and determination of cyanide in properly prepared samples of biological and environmental materials are spectrophotometric (Cruz-Landeira et al. 2000) and chromatographic (Tracqui et al. 2002) methods including gas and liquid chromatography (Table 8).

**Table 8:** Preparation and analytical techniques for cyanide determination

Analytical technique	Metrological parameters	Type of sample	References
IC-PAD	Linearity 0.0147–2.45 μg/mL	Mainstream smoke	Zhang et al. (2011); Xu et al. (2006); Wu et al. (2015)
	LOD 1–3 μg/mL	Drinking water	
	Recovery 94.3–101%	Liquor	
SI-GD	Linearity up to 200 g/L	Mineral water	Themelis et al. (2009)
	LOD 2.5 μg/L		
	LOQ 7.5 μg/L		
GC			
NPD	LOD 0.003 μg/mL–0.5 μg/L	Petrochemical wastewater	Scheneider et al. (1997); Felby (2009)
	Recovery 76.8–121.5%	Blood	
FTD	MDL 0.021 ppbv	Air (lower atmosphere)	Ambose et al. (2012)
RGD	LOD 30 ppt	Air (stratosphere)	Scheneider et al. (1997)
MS	LOD 0.01–0.2 μg/mL Recovery 80%	Mainstream smoke	Marcilla et al. (2012); Moriya and Hashimoto (2003); Liu et al. (2009a); Tsunge et al. (2000)
		Blood	
		Urine	
		Saliva	
μECD	Recovery 86–116%	Mainstream smoke	Xu et al. (2006)
	LOD 0.6 ng/mL		
	Linearity 0.0250–15.0 ng/mL		
MS			
SFIT	LOD 1 ppb	Air (atmosphere) Breath	Zhao et al. (2000); Španěl et al. (2007a, b); Dummer et al. (2013)
PTR-TOF	–	Engine exhaust	Moussa et al. (2016)
MS/MS			
ESI	LOD 0.001 μg/mL Recovery 96–117%	Urine Gastric content	Minakata et al. (2009)
LC	LOD 0.5 ng/mL Linearity 0.0024–0.331 ng/mL	Mainstream smoke Grapevine (leaf)	Mottier et al. (2010); Franks et al. (2005)
Polarography	–	Mainstream smoke	Mahernia et al. (2015)
Spectrophotometric method	Recovery 97–109% LOD 0.007 μg/mL–0.02 mg/mL *λ* = 310–578 nm	Electroplating wastewater Wastewater Drinking water Soil Engine exhaust Mainstream smoke Seeds Leaf Flour Cassava pulp Blood Urine Nasal discharge Breath	Karlsson and Botz (2004); Ashley et al. (2014); Absalan et al. (2010); Abassi et al. (2010); Dadfarnia et al. (2007); Hassan et al. (2007); (Matsumura and Kojima (2003); Mansfeldt and Biernath (2000); Manar et al. (2011); Shehong et al. (2005); Rennert and Mansfeldt (2006); Ma et al. (2010); Abdullah et al. (2013); Surleva and Drochioiu (2013); McAllister et al. (2011)
HPLC-UV	LOD 0.1 μg/mL Recovery 98%	Seed Blood	Bolarinwa et al. (2015); Bolarinwa et al. (2014)
Capillary electrophoresis/UV spectrometry	LOD 0.002 μg/mL Recovery 92–106%	Urine Saliva	Zhang et al. (2015); Jermak et al. (2006)
Electrochemical method	LOQ 0.10 mg/L	Blood	Ferrari et al. (2001)
GFIT	–	Savannah fire	Paton-Walsh et al. (2010)
The AOAC quantitative titrimetric method	–	SEED	Chove and Mamiro (2010)
Dräger gas detection tube	–	Air in car	Mangnusson et al. (2012)
CIMS	LOD 37 pptv	Air (stratosphere)	Viggiano et al. (2003)
IMRMS	–	Air (stratosphere)	Singh et al. (2003)

Cyanide ions in plants, water, soil and air occur in many forms of compounds. Cyanogenic glycosides can be determined by a variety of chromatographic techniques, where the main advantage is analysis of primary forms of such glycosides; however, they are relatively expensive. An indirect method of cyanogenic glycoside determination is based on the determination of hydrogen cyanide after acid or enzyme hydrolysis.

Beside the spectrophotometric and chromatographic techniques, chemiluminescence (Goi et al. 2007) or capillary electrophoresis is used, however not so often, in the analysis of environmental samples (Fasco et al. 2007; Sadeg and Belhadj-Tahar 2009). Mass spectrometry with ionization of selected ions in stream (SIFT-MS) is used particularly in the determination of HCN in the exhaled air. Atomic absorption spectroscopy technique cannot be directly applied to the determination of cyanide. However, after applying a microcolumn saturated with ionic silver, it was possible to use FI-FAAS techniques for analysis of cyanide in samples of wastewater (Dadfarnia et al. 2007).

In biological samples, due to the short half-life of cyanides, which ranges from several minutes to few hours at most, often their concentration is determined indirectly by determining the concentration of one of their metabolites or CN-protein adducts. Determination of cyanide ions in biological samples is possible by prior cyanide distillation or microdiffusion to solution of an absorbent material. Then, spectrophotometric methods are used for analysis of cyanide ion. The method is based on the König reaction, where the cyanide anion is oxidized with chloramine-T to cyanogen halide, which is the most accurate colorimetric method. Spectrophotometric method is a universal one; however, the limit of detection at the level of milligrams per litre (or mg/kg) narrows its usage (Goi et al. 2007).

In contrast to spectrophotometric techniques, chromatographic techniques are characterized by a low limit of detection at the level of milligrams per litre and high precision. Depending on the type of detector, gas chromatography is used to analyse various samples: neurophysiological detector (NPD) and FID for water and industrial wastewater (Wan et al. 2015), MS for biological materials (Torikaiu et al. 2005) and μECD for air tobacco smoke (Akintonwa et al. 1994). However, unlike the GC-FID, analytes present in the sample are examined by a GC-NPD method and they require derivatization phase (Wan et al. 2015). Nonetheless, the widest range of concentrations (0.05–10 μg/mL) can be attributed to gas chromatography mass spectrometry, while the lowest limit of detection is typical for capillary electrophoresis technique combined with UV detection. Electrochemical techniques and ion chromatography are characterized by high sensitivity and low detection limits (1 μg/L). Electrochemical methods have been used for determination of HCN in exhaled breath and blood. The versatility of this method causes its extensive use (Giuriati et al. 2004; Christinson and Rohrer 2007).

## Conclusions

The presence of cyanide ions in food and their use in the industry are dangerous to people’s health and safety. Compounds containing cyanide ions are rapidly acting poison, which mainly interferes with the process of cellular respiration, that results in a number of ailments and illnesses and even death. Because of the cyanide ion toxicity, especially important is their determination in environmental and biological samples. The development of procedures to enable quantitation of these ions in environmental samples and in samples of biological materials allows the assessment of risks resulting from human exposure to the cyanide ions in the work environment in food and in the air.

One of the most important aspects of the cyanide ion analysis is the step focused on preparing samples for analysis. It is related to the fact that cyanide ions are not stable ones, and they occur in various forms. The presence of matrix interferences must be also considered in the preservation procedure. Sulphides and reduced sulphur compounds interfere through formation to thiocyanate. Sulphite reacts with strong cyanide complexes at pH >10, decreasing the cyanide concentration. Oxidants such as residual chlorine or hydrogen peroxide are known to interfere. If sample contain oxidants, add a reducing agent. Sodium arsenite (NaAsO_2_) and sodium thiosulphate (Na_2_S_2_O_3_) are preferred reducing agents. Most cyanide analysis sampling protocols specify the preservation of samples at a pH of 12 or higher. During the preparation of environmental samples, extraction techniques (LLE and GLE) are used for cyanide ion determination while filtration and centrifugation are used in the case of biological samples.

Recently, in the literature, information can be found on the use of samples of biological and environmental materials in the cyanide analytics (Table 9). Especially interesting are biological materials, due to the effect of cyanide on human health and life.

**Table 9:** Application of analytical techniques for the determination of cyanide in various samples

Determination technique	Type of matrix					
	Water	Wastewater	Food	Air	Soil	Biological materials
Chromatography						
Gas		+		+		+
Liquid			+	+		+
Ion	+		+	+		
Spectrophotometric	+	+	+	+	+	+
Others						
SI-GD	+					
SFIT				+		
PTR-TOF				+		
ESI						+
Polarography				+		
CE						+
IMRMS				+		

When looking at information on the used analytical techniques, it can be noticed that the most interesting one became gas chromatography liquid and ion chromatography, which allow to achieve lower limits of quantification (1 μg/L); furthermore, they are characterized by good selectivity and reproducibility. In relation to the increased interest in the subject of cyanide ion analytics, researches aim to use other analytical techniques. It is, however, necessary to carry out validation on real samples.
